# An Update on Coronary Artery Disease and Chronic Kidney Disease

**DOI:** 10.1155/2014/767424

**Published:** 2014-03-10

**Authors:** Baris Afsar, Kultigin Turkmen, Adrian Covic, Mehmet Kanbay

**Affiliations:** ^1^Department of Nephrology, Numune State Hospital, 42690 Konya, Turkey; ^2^Department of Medicine, Division of Nephrology, Mengucek Gazi Training and Research Hospital, Erzincan University, Erzincan, Turkey; ^3^Nephrology Clinic, Dialysis and Renal Transplant Center, C.I. Parhon University Hospital, “Gr. T. Popa” University of Medicine and Pharmacy, 700115 Iasi, Romania; ^4^Department of Medicine, Division of Nephrology, Istanbul Medeniyet University, Istanbul, Turkey

## Abstract

Despite the improvements in diagnostic tools and medical applications, cardiovascular diseases (CVD), especially coronary artery disease (CAD), remain the most common cause of morbidity and mortality in patients with chronic kidney disease (CKD). The main factors for the heightened risk in this population, beside advanced age and a high proportion of diabetes and hypertension, are malnutrition, chronic inflammation, accelerated atherosclerosis, endothelial dysfunction, coronary artery calcification, left ventricular structural and functional abnormalities, and bone mineral disorders. Chronic kidney disease is now recognized as an independent risk factor for CAD. In community-based studies, decreased glomerular filtration rate (GFR) and proteinuria were both found to be independently associated with CAD. This paper will discuss classical and recent epidemiologic, pathophysiologic, and clinical aspects of CAD in CKD patients.

## 1. Introduction

Chronic kidney disease (CKD) is associated with accelerated cardiovascular risk. Data from prospective studies support that cardiovascular diseases (CVD) remain the most common cause of morbidity and mortality in patients with CKD and end-stage renal failure (ESRF) receiving hemodialysis (HD) and peritoneal dialysis (PD) [1]. The spectrum of CVD not only involves obstructive coronary artery disease (CAD), but also involves other disease states such as chronic heart failure, sudden death, and arrhythmias. The main factors for the heightened risk in this population, beside advanced age and a high proportion of diabetes and hypertension, are malnutrition, chronic inflammation, accelerated atherosclerosis, highly prevalent endothelial dysfunction (ED) and coronary artery calcification (CAC), left-ventricular structural and functional abnormalities, and bone mineral disorders (BMD) [2–4]. Chronic kidney disease is now recognized as an independent risk factor for CAD in community-based studies as well as in high cardiovascular (CV) risk populations. In community-based studies, decreased glomerular filtration rate (GFR) and proteinuria were both found to be independently associated with CAD [5–7]. The risk for coronary artery disease (CAD) increases gradually with the decline of glomerular filtration rates; that means that end-stage renal failure (ESRF) patients have the highest CVD risk among CKD population [8–11]. Thus by the light of these aforementioned data, this paper will discuss classical and recent epidemiologic, pathophysiologic, and clinical aspects of CAD, especially focusing on obstructive CAD disease in CKD patients.

## 2. Epidemiology

The relationship between CV events and CKD has been repetitively shown by the epidemiologic studies. The largest population-based study done by Go et al. demonstrated that a decline in GFR was the main independent risk factor for CV events, including hospitalization secondary to peripheral artery disease (PAD), CAD, congestive heart failure (CHF), or stroke even after the elimination of confounding risk factors, in more than 1.1 million adults [12]. Similar findings were also reported in a systematic review considering approximately 1.4 million adults from 42 different cohorts [13]. According to this paper's results, the risk of all-cause mortality was the highest in patients with lowest baseline GFR and vice versa. The gradual fall of GFR was also found to be associated with a gradual increase of death. Cardiovascular risk increased even in the early stages of CKD, particularly in the elderly. In a study including approximately 30.000 older CKD patients with estimated GFR of less than 90 mL/min/1.7 m^2^, the rate of mortality at five years was 19.5%, 24.3%, and 45.7% in those with CKD stages 2, 3, or 4, respectively [14]. Additionally, elderly HD patients have also increased risk acute coronary event and mortality after acute coronary event. In one study, it was demonstrated that elderly HD patients (>65 years old) have an odds of 3.289 for acute coronary syndrome and odds of 1.693 for death [15].

Considering the depth and the quality of the epidemiological evidence, to date, both the American College of Cardiology/American Heart Association (ACC/AHA) and the National Kidney Foundation (NKF) recommend that CKD should be considered as equivalent of CAD [16, 17].

## 3. Pathophysiology of CAD in CKD

Atherosclerosis is a condition characterized with formation of plaques on the intimal layer of vessels. According to the AHA guidelines, coronary atherosclerotic plaques constitute most of the CVD in general population [18]. However, the pathophysiology of vascular disease in CKD is quite different from that related to atherosclerosis, in the general population [19]. Beside traditional risk factors including hypertension, diabetes, dyslipidemia, and advanced age, novel risk factors such as endothelial dysfunction (ED), CKD-MBD abnormalities (hyperphosphatemia, hyperparathyroidism, and vascular calcifications), increased oxidative stress, and inflammation are highly prevalent and seem to play a more important role for vascular disease in CKD and ESRF patients compared to healthy subjects [4, 20–22]. Several studies demonstrated that systemic persistent inflammation particularly could be the main factor responsible for this increased risk in ESRF patients regardless of the renal replacement therapy [23]. To prove this hypothesis, several biomarkers including C-reactive protein, interleukin (IL)-1*β*, IL-6, and tumor necrosis factor-*α* (TNF-*α*) were considered in CVD and CKD populations [23, 24].

Apart from the factors mentioned above, why the CKD patients are more prone to worse CV outcomes is still unclear. In the general population, many patients with CAD develop coronary collateral circulation to overcome obstruction of the atherosclerotic coronary arteries. Charytan et al. hypothesized that CKD patients might have less collateral blood supply to ischemic area of the myocardium and this hypothesis might partially explain why CKD patients have much worse CV outcomes and death. However, this study failed to prove this hypothesis because both CKD patients and subjects without CKD had similar culprit artery collateral supply (25% versus 27.2%, resp., *P* = 0.76) [25].

The influence of vascular calcification (VC) deserves special mention in this context. Vascular calcification is very common and is becoming more prevalent with the worsening of kidney function in patients with CKD and ESRF. The importance of this process has been demonstrated by the tight relationship between VC and increased cardiac mortality in this population [26]. The hemodynamic consequences of VC include a decrease in coronary microcirculation and arterial elasticity, an increase in pulse wave velocity, and increased left ventricular hypertrophy [26, 27]. Vascular calcification may develop in the intimal or the medial layer of the vessel wall. The latter is also named as “Monckeberg's sclerosis” which is much more common in patients with CKD compared to general population [27]. The main differences between these two types of VC are as follows: (i) intimal calcification is highly associated with inflammation and focal occlusion secondary to the plaque formation; however, medial calcification is characterized by diffuse pipe type calcification of muscular arteries, and (ii) intimal calcification is commonly seen in coronary, carotid arteries, and aorta, whereas medial calcification is commonly observed in tibial and femoral arteries [28–30]. Malnutrition-inflammation-atherosclerosis/calcification (MIAC) syndrome has been defined as the interaction between increased levels of proinflammatory cytokines, malnutrition, and atherosclerosis/calcification in ESRF patients [31, 32]. Stenvinkel et al. [33] hypothesized that malnutrition, inflammation, and atherosclerosis cause a vicious cycle, and that proinflammatory cytokines play a central part in this process. The presence of MIAC components was found to be associated with increased mortality and morbidity in ESRF patients receiving PD [33] or HD [34]. The coronary artery calcification is a part of the extended state of vascular calcification which can be detected even in the early years on dialysis [24, 29] that reflects the severity of atherosclerotic vascular disease and predicts cardiovascular events [35, 36]. Wang et al. showed an important association between malnutrition-inflammation-atherosclerosis and valvular and vascular calcification in PD patients [32]. In a recent study, the correlation of coronary artery calcification score (CACS) with coronary flow velocity reserve (CFR) was investigated in HD patients [37]. According to the results of this study, HD patients with CACS >10 had a significantly lower CFR and the functional deterioration of coronary arteries started from low levels of CACS.

Epicardial adipose tissue (EAT) is the true visceral fat depot of the heart that accounts for approximately 20% of total heart weight and covers 80% of the cardiac surfaces and is mostly in the grooved segments along the paths of coronary arteries [38–40]. Recent studies showed a close relationship between CAD and EAT using multidetector computed tomography (MDCT) and echocardiography in healthy subjects and patients at a high risk of CAD [41–44]. Although the pathophysiologic role of EAT is not clear to date, the researchers suggest that EAT may act as an extremely active organ that produces several bioactive adipokines as well as proinflammatory and proatherogenic cytokines such as TNF-*α*, monocyte chemotactic protein (MCP-1), IL-6, and resistin [43, 45–48]. In a recent study, we demonstrated the relationship between MIAC syndrome and EAT in ESRF patients receiving HD or PD [49]. Taken together, these factors may contribute to premature CVD and the markedly increased mortality in patients with ESRF. Treatment of CAD in patients with CKD is beyond the scope of this paper.

## 4. Clinical Studies of CAD in CKD Patients

In a substudy of Acute Catheterization and Urgent Intervention Triage strategY (ACUITY) trial, Acharji et al. [50] aimed to show the prognostic value of baseline troponin levels of 2179 CKD patients with moderate and high risk of ACS. Of 2179 CKD patients, 1291 had elevated baseline troponin (59.2%). CKD patients with higher baseline serum troponin levels had significantly higher rates of death, MI, or unplanned revascularization at 30 days and 1 year, compared with CKD patients without baseline troponin elevation. Another important result of this study confers that baseline elevation of troponin independently predicts death or MI at 30 days and 1 year (HR = 2.05 (1.48–2.83), *P* < 0.0001 and HR = 1.72 (1.36–2.17), *P* < 0.0001, resp.). However, diagnosis of ACS in the patients with CKD based on troponin levels should be interpreted cautiously.

In general, cardiac mortality has been estimated as 40% in dialysis patients [51]. Additionally, this high rate reaches up to 50% in diabetic ESRF patients without any ACS symptoms. Accurate diagnosis of ACS is quite different in this population. In an observational study, the authors investigated the clinically different parameters after myocardial infarction in CKD (baseline serum creatinine ≥2.5 mg/dL), non-CKD, and dialysis patients. There were 2390 dialysis patients, 29319 advanced CKD patients, and 274,777 non-CKD patients [52]. On admission, chest pain was seen in 41.1%, 40.4%, and 61.6% of dialysis, advanced CKD and non-CKD patients, respectively. On the other hand, ST elevation was seen in only 17.6% of dialysis patients, in 15.9% of advanced CKD patients, and in 32.5% of non-CKD patients. Lastly, in hospital, mortality was 21.7% for dialysis patients, 23.0% for non-CKD patients (the authors attribute this effect to older age), and 12.6% for non-CKD patients. As a take-home message, the authors suggested that classical symptoms and signs of myocardial infarction may not be observed in CKD and dialysis patients. Besides, advanced CKD patients with AMI are high-risk, clinically similar to dialysis patients with AMI [52].

Presence of hypertension in the patients with chronic kidney disease is one of the most important contributing factors for CV morbidity and mortality. For detecting hypertension, ambulatory blood pressure monitoring (ABPM) is one of the important diagnostic tools especially in patients with poorly controlled hypertension [53]. Andersen et al. showed that approximately 30% of CKD patients had office BP measurements higher than ABPM, whereas approximately 28% of the patients normal Office BP measurements but higher ABPM [54]. ABPM showed a strong correlation with left ventricular mass index (LVMI) [55] and proteinuria [56], compared to single casual office BP measurements in patients with CKD. A study comparing the prognostic value of office BP and home BP monitoring showed that home measurements were superior to office BP and predicted ESRF independently of other risk factors [57]. In hypertensive CKD patients, inappropriate left ventricular hypertrophy may occur which can be estimated by the ratio of observed to predicted left ventricular mass (LVM). Recently, the ratio of observed to predicted LVM was found to be independently associated with increased CV events in patients with CKD stages 3–5 [58].

## 5. Prognosis of CAD in Patients with CKD

CKD patients with CAD have a worse prognosis than patients with preserved kidney functions. In-hospital complication rates and long-term mortality were found to be highest among patients receiving HD [59]. Previous studies showed that there was an adverse association between GFR decline and cardiovascular prognosis even in CAD patients who underwent percutaneous coronary intervention [60] or coronary artery bypass grafting [61]. The proposed explanations of this poor prognosis are presence of hypertension, diabetes, advanced age, hypervolemia, decreased glomerular filtration rate, proteinuria inflammation, oxidative stress, and so forth [62, 63]. Thus, the detrimental effects of these risk factors may explain the heightened mortality rates in this population.

## 6. Treatment Strategies for CAD in CKD

### 6.1. Medical Therapy

Standard cardiovascular medications including aspirin, beta blockers, angiotensin converting enzyme inhibitors, and statins are considerably underprescribed in CKD patients [64, 65].

Regarding the statins, while observational studies have suggested a beneficial role in ESRF [66, 67], the randomized double blind 4D trial [68] detected no reduction in the composite endpoint of CV death, stroke, or nonfatal MI in 1,255 patients with type II diabetes and ESRF on dialysis, assigned to atorvastatin 20 mg versus placebo. Furthermore, the recent randomized double-blind AURORA trial demonstrated no difference in composite primary endpoint of CV death, stroke, or nonfatal MI over 3.8 years in 2,776 dialysis patients receiving rosuvastatin 10 mg versus placebo [69], raising uncertainty over the benefit of statins in patients with ESRF.

Lastly, the Study of Heart and Renal Protection (SHARP) trial [70] is worthy to mention. This randomized double-blind trial included 9270 patients both with chronic kidney disease and ESRF on hemodialysis and peritoneal dialysis (3023 on dialysis and 6247 not) with no known history of myocardial infarction or coronary revascularisation. Patients were randomly assigned to simvastatin 20 mg plus ezetimibe 10 mg daily (4650 patients) versus matching placebo (4620 patients). The key prespecified outcome was the first major atherosclerotic event (nonfatal myocardial infarction or coronary death, nonhemorrhagic stroke, or any arterial revascularisation procedure). All analyses were by intention to treat. After 4.9 years of followup, there was a 17% proportional reduction in major atherosclerotic events in simvastatin plus ezetimibe versus placebo; rate ratio [RR] was 0.83, 95% CI 0.74–0.94; log-rank *P* = 0.021, and there were significant reductions in nonhemorrhagic strokes (131 [2.8%] versus 174 [3.8%]; RR 0.75, 95% CI 0.60–0.94; *P* = 0.01) and arterial revascularisation procedures (284 [6.1%] versus 352 [7.6%]; RR 0.79, 95% CI 0.68–0.93; *P* = 0.0036) in simvastatin plus ezetimibe group. On subgroup analysis, however, the investigators did not observe a clinically or statistically significant reduction in either mortality or the cardiovascular event rate in the dialysis population given active treatment compared with those on placebo (15% versus 16.5%, resp.). Consequently, the results of the SHARP study for patients on dialysis were similar to those from the AURORA and 4D studies.

Apart from their effects on ischemia beta blockers have antifibrillary activity, sympathetic inhibitory effect, decrease in frequency of ventricular arrhythmia, and increase in baroreflex sensitivity [71]. Surprisingly, however, few studies were conducted to demonstrate the effect of beta blockers in CKD patients. It is also uncertain that all beta blockers are equal in their action in CKD patients. One study examined the influence of beta blocker usage on the increased cardiovascular risk associated with CKD among a large cohort of male and female patients who are not on dialysis and with established coronary artery disease (CAD). They showed that beta blockers are associated with a reduced risk of acute myocardial infarction or sudden cardiac death in patients with CAD irrespective of kidney function when compared to CAD patients without kidney impairment and not receiving beta blockers. The relative reduction in the incidence of the primary end-point by beta blockers was somewhat better for patients with relatively preserved kidney function. Furthermore, the observed relationship remained significant after multivariate analysis [72]. A study by Pun et al. [73] revealed that beta blockers increased the odds of surviving after cardiac arrest. They demonstrated that, in 729 patients who were identified as having a confirmed in-center cardiac arrest, the 24-hour survival rate is only positively related to beta blocker usage after adjusting for covariates (RR 0.61, 95% CI 0.44–0.86, *P* = 0.005). Cice et al. [74] randomized 114 HD patients with dilated cardiomyopathy to receive carvedilol or placebo in a controlled study. Of note, all patients were either on angiotensin-converting enzyme inhibitors (ACEi) or angiotensin-receptor blockers (ARB) and were symptomatic for heart failure (New York Heart Association Functional Class II-III). Additionally, 68% had a history of ischemic heart disease (defined as a documented history of myocardial infarction typical angina, an exercise electrocardiogram positive for ischemia, or angiographic evidence of coronary disease). After 12-month treatment with carvedilol, there was a statistically significant improvement in the left ventricular ejection fraction (from 26 to 36%). Then they followed the same patient population for an additional 12-month period. After 2-year followup, they demonstrated that 51.7% of the patients died in the carvedilol group compared to 73.2% in the placebo group (*P* < 0.01). There were significantly fewer all-cardiovascular deaths (29.3 versus 67.9%, *P* < 0.0001) among patients receiving carvedilol than among those receiving placebo. Although these results of beta blockers on chronic dialysis patients were encouraging, they are relatively underused in CKD patients. In a recent paper, it was recommended that especially some beta blockers with approved indications for cardiovascular mortality benefit such as metoprolol and carvedilol should be more frequently used in CKD patients [75]. Although the favorable effects of ACEi in patients without renal disease have been shown in multiple studies, the data about the use of ACEi in dialysis patients with heart failure or their effect on SD are scarce. Also, the effect of ACEi treatment may be different in specialized population groups (e.g., dialysis patients). For example, in one study, it was reported that, in contrast to the general population, ACEi treatment reduced heart rate variability (HRV) in ESRF patients. The authors of this study suggest that the risk versus benefit of ACEi use in patients with ESRF warrants further investigation [76]. In a retrospective study, Efrati et al. reported a 52% reduction in mortality among dialysis patients on ACE inhibitors despite no difference in blood pressure reduction [77]. On the contrary, a prospective trial of fosinopril in dialysis patients did not demonstrate a significant difference in the rate of major cardiovascular events [78]. There is paucity of data related to the use of ARBs for the prevention of cardiac mortality in dialysis patients. A small randomized trial of candesartan in dialysis patients demonstrated an almost 3-fold reduction in cardiovascular events and a reduction in number of fatal arrhythmias. However, the significance of the latter finding is limited by the small number of events [79]. In patients with a history of cardiac arrest, Pun et al. demonstrated that the use of ACEi/ARBs was associated with better survival at 6 months after adjusting for various covariates (OR 0.51, 95% CI 0.28–0.95, *P* = 0.03) [73]. There is no doubt that larger trials will be necessary before any strong conclusions on the use of ACE inhibitors or ARBs to prevent SD in HD patients can be made.

### 6.2. Revascularisation

Revascularization is indicated for relief of anginal symptoms, prognostic benefit, or both. While considering revascularization, one should consider two aspects: the application rate of optimal medical therapy and the efficacy and risks of revascularization [80].

#### 6.2.1. Percutaneous Coronary Intervention

The percutaneous coronary intervention can be divided into two phases: one is prestent period and the other is stent period. Many studies performed in the prestent era reported unfavorable outcomes with balloon angioplasty in CKD [81–84], unacceptable high rates of procedural complications (~10%) and restenosis (~80%) which were postulated to relate to high prevalence of diffusely diseased small vessels, extensive vascular calcification, diabetes, and increased prothrombotic activity [85–87]. However, the introduction of coronary stents has improved both early success rates and long-term outcomes. One study demonstrated equivalent procedural success, inhospital mortality, stent thrombosis, and one-year clinical restenosis rates in dialysis patients compared to those with normal renal function undergoing PCI with stents [88]. In the PRESTO trial, a prospective randomized trial of over 11,000 patients undergoing mainly single-vessel stenting, 1,749 patients with eGFR < 60 mL/min/1.73 m^2^, and 4,054 with GFR 80–89 mL/min/1.73 m^2^ was identified. Rates of major adverse cardiac events at 30 days were low (1.5%) and did not differ by eGFR. Importantly, the groups with lower eGFR had no excess in angiographic restenosis or event rates at nine months [89]. With respect to drug-eluting stents (DES) one study has shown that, in CKD patients, treatment with DES compared with bare-metal stents resulted in lower nine-month angiographic restenosis rates (2.1% versus 20.5%, *P* < 0.01) and one-year target vessel revascularization rates, that is, subsequent requirement for further revascularization in the same vessel (3.3% versus 12.2%, *P* < 0.001). However, the occurrence of death, MI, and stent thrombosis at one year was similar in both groups, independent of renal function [90]. However, other studies have shown that decreased renal function itself has a bad prognostic sign. Blackman et al. showed that the composite endpoint of inhospital death, MI, and revascularization was inversely related to baseline renal function [91]. In another study, DES showed that estimated EGFR was an independent predictor of stent thrombosis [92].

#### 6.2.2. Coronary Artery Bypass Grafting

Although cardiovascular diseases are common in CKD patients, there is a tendency to perform less invasive procedures including coronary artery bypass grafting (CABG) in these patients. Thus relatively few studies are available which compare bypass grafting to medical therapy. The major reason for this is that CABG is associated with high levels of procedural risk and postoperative complications in patients with CKD and ESRF. Liu et al. demonstrated that HD patients undergoing CABG had higher mortality and mediastinitis rates [61]. Of course this does not mean that medical therapy is better than CABG in ESRF patients. Rahmanian et al. also demonstrated that mortality was higher in HD patients undergoing CABG [93]. These worse outcomes have also been shown in CKD patients who are not on dialysis, and renal function has been shown to have an independent prognostic impact after CABG [94–96]. Still, there are studies comparing medical and stent treatment to CABG in CKD population. In a very large study, Hemmelgarn et al. demonstrated that CABG was superior to medical therapy in CKD and dialysis patients. Additionally, compared to PCI, CABG was associated with a significantly lower risk of death in the CKD group but not in the dialysis group [97]. Concerns regarding the risks of surgical manipulation of a calcified aorta, and the potential risks related to the extracorporeal circulation, have prompted investigation into the potential benefit of offpump CABG surgery in CKD patients. While some studies have suggested lower mortality by offpump modality [98], others reported better long-term survival in dialysis patients who underwent onpump CABG despite the fact that the offpump group had lower operative mortality [99]. These studies consistently demonstrated CABG is better compared to stents [100–102]. However, these results should be interpreted with caution due to small populations and difficulty in controlling for confounders including comorbidity and degree of coronary disease, as well as selection bias. [103].

In one prospective study, patients with CKD are randomized to CABG versus stent placement. There was no difference with respect to mortality, MI, or stroke although repeated revascularization was higher in the stent group [104]. These findings were confirmed later by Aoki et al. [105]. With respect to DES and CABG in CKD patients, the results were similar and there was no difference with respect to death, MI, or stroke; however, revascularization rates were higher in patients with DES treatment [106].

## 7. Conclusion

The risk of CAD was unexpectedly high in patients with CKD and ESRF. Besides, the traditional risk factors such as advanced age and a high proportion of diabetes and hypertension, novel risk factors commonly seen in CKD patients including chronic inflammation, oxidative stress, and hyperparathyroidism might provoke the underlying pathophysiological mechanisms of CAD. Revascularization in CKD and HD patients is more complicated and has more side effects compared to normal population. Obviously, prospective randomized data are needed to compare the effects of treatment strategies including medical treatment and stent placement CABG in these patients.
